# Quantifying the reliability gap in cross-domain plant disease classification: benchmarking the limited efficacy of standard mitigation techniques under controlled-to-field shift

**DOI:** 10.3389/fpls.2026.1826962

**Published:** 2026-05-22

**Authors:** Kun Xiang, Danxi Shi, Xiangbo Zhu

**Affiliations:** 1 Research Center of Machine Learning and Environment Science, China Three Gorges University, Yichang, China; 2 Plant Protection Research Center, Shenzhen Polytechnic University, Shenzhen, China

**Keywords:** backbone comparison, confidence calibration, cross-domain reliability, domain shift, ensemble uncertainty, plant disease diagnosis, selective prediction

## Abstract

**Introduction:**

Confidence calibration, selective prediction, out-of-distribution scoring, and deep ensembles are mature techniques in machine learning, yet their efficacy under the severe domain shift encountered when plant disease classifiers move from controlled laboratory imagery to heterogeneous field photographs has not been systematically benchmarked.

**Methods:**

Models trained on PlantVillage were evaluated on PlantDoc leaf-level crop images under a parent-image-aware split protocol, and a suite of standard mitigation techniques was applied to characterize the reliability gap. Analyses included temperature scaling and selective prediction for a fine-tuned ResNet-50, quantitative image-level shift analysis, Grad-CAM visualization, simple target-aware adaptation baselines, frozen-feature backbone comparisons, and ensemble baselines.

**Results:**

In the primary case study, a fine-tuned ResNet-50 suffered a 67.7-percentage-point accuracy collapse upon cross-domain transfer, while mean predicted confidence remained at 79.76%. *Post-hoc* temperature scaling reduced calibrated ECE to 0.3645 but left selective risk at 80% coverage at 64.30%. Quantitative image-level shift analysis confirmed large-effect-size differences in saturation (*d* = 3.90), border edge density (*d* = 3.33), and foreground-occupancy proxy (*d* = 2.48) between the two domains, while Grad-CAM visualizations showed that the model shifts attention from lesion-centered regions in PlantVillage to background-dominated areas in PlantDoc. Simple target-aware mitigations, including adaptive batch normalization and feature moment matching, improved accuracy from 0.321 to 0.343 and 0.366, respectively, whereas DANN-style adversarial adaptation degraded performance to 0.252. A frozen-feature backbone comparison across five backbones showed that, within the energy-scoring frozen-backbone comparison, DINOv2-S/14 achieved the highest unknown-detection AUROC (0.764) and the lowest selective risk at 80% coverage (0.520), with paired Wilcoxon tests confirming statistically significant accuracy and macro-F1 differences across backbones. Two ensemble baselines were evaluated: a warm-start end-to-end ResNet-50 ensemble reduced calibrated ECE to 0.063 but achieved only 0.666 AUROC, while a lightweight DINOv2 linear-probe ensemble achieved 0.779 AUROC after calibration but under limited epistemic diversity.

**Discussion:**

Neither ensemble established deployment-grade reliability: the best selective risk at 80% coverage across all configurations remained above 0.51. The principal contribution is a reproducible, deployment-oriented reliability characterization showing that standard *post-hoc* and lightweight adaptation techniques reduce but do not eliminate the severe reliability gap under controlled-to-field transfer in agricultural computer vision.

## 1 Introduction

Crop diseases inflict global yield losses estimated at 20–40% annually (Savary et al., 2019), threatening food security for billions and imposing acute economic hardship on smallholder farmers who lack timely access to expert diagnosis. Over the past decade, deep convolutional neural networks have attracted intense interest as potential tools for automated disease recognition (Boulent et al., 2019; Saleem et al., 2019), with headline accuracies frequently exceeding 95% on benchmark datasets such as PlantVillage (Mohanty et al., 2016; Sladojevic et al., 2016; Brahimi et al., 2017; Ferentinos, 2018; Too et al., 2019). These results have fueled expectations that smartphone-based diagnostic applications could democratize plant pathology expertise (Johannes et al., 2017), delivering real-time guidance to farmers regardless of their proximity to extension services. More broadly, the convergence of deep learning with Internet of Things (IoT) platforms and precision agriculture workflows—including context-aware fertilizer recommendation systems (Khan et al., 2022a), intelligent evapotranspiration optimization for precision irrigation and saline-soil reclamation (Khan et al., 2022b; Bashir et al., 2023), and lightweight crop monitoring frameworks based on modern optimization (Ramu et al., 2026)—has intensified the demand for models whose outputs can be trusted not only by human interpreters but also by automated decision pipelines that act on predictions without expert review. Convolutional neural network approaches have further advanced automated disease detection from leaf images (Vishnoi et al., 2023), yet the reliability of such models under realistic deployment conditions remains largely unexamined.

A growing body of evidence, however, suggests that the gap between benchmark performance and field-level reliability is far wider than accuracy figures alone convey. PlantVillage images are captured under tightly controlled conditions—uniform backgrounds, consistent illumination, single centered leaves—whereas photographs taken in working fields contain cluttered canopies, variable sun angles, overlapping foliage, motion blur, and insects or soil particles that occlude symptoms. This discrepancy constitutes a well-documented instance of domain shift (Shimodaira, 2000; Ben-David et al., 2010): the statistical distribution on which the model was trained diverges substantially from the distribution it encounters at deployment. Prior studies have reported accuracy drops of 20 to 50 percentage points when models trained exclusively on PlantVillage are evaluated on field-sourced datasets such as PlantDoc (Ramcharan et al., 2017; Barbedo, 2018; Arsenovic et al., 2019), confirming that high in-domain accuracy is necessary but far from sufficient for practical utility.

What has received considerably less attention is a subtler and arguably more dangerous facet of this shift: the behavior of a model’s confidence scores when it is wrong. Modern deep neural networks are known to produce poorly calibrated probability estimates even within their training domain (Niculescu-Mizil and Caruana, 2005; Guo et al., 2017), and the problem intensifies under distribution shift (Ovadia et al., 2019). A classifier may assign a probability of 0.92 to an incorrect diagnosis, giving the end user—farmer, agronomist, or downstream decision support system—no reliable signal that the output should be questioned. In medical diagnostics the consequences of such overconfidence have been extensively discussed (Jiang et al., 2012; Rączkowska et al., 2019); in agriculture the stakes are analogous. Misidentifying a fungal infection as a nutrient deficiency could lead to inappropriate chemical application, delayed treatment, unnecessary cost, and environmental harm. If automated tools are to earn the trust of agricultural practitioners, they must not only classify correctly when they can but also communicate honestly when they cannot.

Meeting this dual requirement calls for two complementary mechanisms. The first is confidence calibration: adjusting output probabilities so that they faithfully reflect empirical correctness likelihood. Temperature scaling—a single-parameter *post-hoc* transformation of the logit vector— has emerged as a simple yet effective baseline (Guo et al., 2017) that preserves classification accuracy while substantially improving the alignment between predicted confidence and observed accuracy. Its simplicity is a practical asset: it requires no architectural modification, minimal additional data, and negligible computational overhead, making it readily applicable to already-deployed models. The second mechanism is selective prediction, or abstention. Rather than forcing the classifier to commit to one of its known categories for every input, selective prediction introduces a policy under which the model may decline to answer when its confidence is insufficient, flagging the sample for human review (Geifman and El-Yaniv, 2017). The resulting trade-off is transparent: higher abstention rates reduce coverage but may also reduce the error rate among the samples that are answered.

A related but distinct risk arises when a model encounters a disease or crop species entirely absent from its training set. In field conditions the closed-world assumption—that every input belongs to one of the known categories—is routinely violated. A tomato early-blight classifier, for instance, may encounter cassava mosaic disease and, lacking any representation of that category, confidently assign it to whichever known class produces the highest activation. Evaluating a model’s ability to reject such unknown-class inputs—studied under the framework of open-set recognition (Bendale and Boult, 2016)—is therefore essential for credible deployment assessment, yet it is rarely reported alongside standard accuracy benchmarks in the plant science literature.

Importantly, the individual techniques employed in this study—confidence calibration via temperature scaling (Guo et al., 2017), *post-hoc* score functions for out-of-distribution detection (Hendrycks and Gimpel, 2017; Liu et al., 2020), selective prediction (Geifman and El-Yaniv, 2017), and deep ensembles for uncertainty decomposition (Lakshminarayanan et al., 2017)—are well-established methods in the broader machine learning literature. Domain adaptation strategies including batch normalization adaptation (Li et al., 2017), feature moment matching, and adversarial domain alignment (Ganin et al., 2016) have likewise been extensively studied. Gradient-weighted class activation mapping (Grad-CAM) (Selvaraju et al., 2017) is a mature interpretability tool, and vision–language pre-training (Radford et al., 2021) has substantially expanded the space of available representations. This paper does not introduce new calibration, scoring, or adaptation algorithms. Its contribution is instead evidentiary: to provide a rigorous, deployment oriented characterization of the severe reliability gap that emerges when plant disease classifiers are transferred from controlled to field conditions, and to benchmark the limited efficacy of these standard mitigation techniques in narrowing that gap.

Despite the maturity of the underlying methods, their empirical behavior under the particular domain shift encountered in agricultural computer vision—from tightly controlled PlantVillage imagery to heterogeneous PlantDoc field photographs—has not been systematically characterized. Existing studies in plant disease recognition tend to focus either on maximizing cross-domain accuracy through domain adaptation or data augmentation, or on uncertainty quantification within a single domain and a single architecture. The intersection of cross-domain transfer, calibration assessment, selective prediction, and open-set rejection, evaluated through the lens of deployment risk rather than benchmark ranking, remains largely unexplored. Moreover, the questions of whether reliability conclusions depend on backbone architecture, whether quantifiable visual distribution differences explain the severity of the gap, and whether standard adaptation or ensemble baselines can close it have not been addressed together in this context.

The present study addresses these gaps through five contributions. First, we provide a quantitative characterization of a severe reliability gap under controlled-to-field transfer, documenting the simultaneous failure of accuracy, confidence calibration, selective prediction, and unknown-class rejection in a fine-tuned ResNet-50 case study evaluated with a parent-image-aware split protocol that prevents data leakage. Second, we explain why the gap is severe by quantifying large-effect-size differences in image-level visual statistics (saturation, edge density, foreground-occupancy proxy) between the two domains and by using Grad-CAM (Selvaraju et al., 2017) visualizations to show that the model shifts attention from lesion-centered regions in source images to background-dominated areas in target images. Third, we benchmark the limited efficacy of standard mitigation techniques—adaptive batch normalization (Li et al., 2017), feature moment matching, and DANN-style adversarial adaptation (Ganin et al., 2016)—showing that simple target-aware methods yield modest improvements but do not establish deployment-grade reliability. Fourth, we extend the analysis across five backbone architectures—ResNet-50 (He et al., 2016), ViT-S/16 (Dosovitskiy et al., 2021), DINOv2-S/14 (Oquab et al., 2024), CLIP ViT-B/16 (Radford et al., 2021), and CLIP ResNet-50—under a standardized frozen-representation protocol, demonstrating that representation quality matters materially but does not close the gap. Fifth, we evaluate two ensemble baselines—a warm-start end-to-end ResNet-50 ensemble and a lightweight DINOv2 linear-probe ensemble—showing that ensemble averaging substantially improves calibration but does not yield the strongest operational performance overall, and that the resulting uncertainty decomposition provides diagnostically useful but limited evidence under the constrained diversity of both ensemble designs.

The remainder of this paper is organized as follows. Section 2 describes the datasets, category alignment, primary model, calibration and selective prediction methods, quantitative shift characterization, Grad-CAM analysis, simple adaptation baselines, backbone comparison protocol, ensemble construction, and evaluation metrics. Section 3 presents empirical results organized around six modules: the severity of the reliability gap in the primary case study, quantitative and visual evidence explaining why the gap is severe, the limited efficacy of simple target-aware mitigations, the effect of stronger representations, ensemble baseline evidence, and a concise cross-method synthesis. Section 4 discusses implications, distinguishes statistical from practical significance, acknowledges limitations, and outlines future directions.

## 2 Materials and methods

### 2.1 Datasets, category alignment, and data partitioning

This study employs two publicly available leaf disease image corpora representing opposite ends of the visual complexity spectrum. PlantVillage, originally assembled by Hughes and Salathé (Hughes and Salathé, 2015) and later expanded by Mohanty et al. (2016), contains over 54,000 photographs of individual leaves captured against uniform backgrounds under controlled, diffuse illumination. The dataset spans 14 crop species and 38 disease or healthy-state categories. Because each image is tightly framed on a single leaf with minimal background clutter, intra-class variability is low and classification accuracy on held-out splits routinely exceeds 95% in published work. In the present study, PlantVillage serves exclusively as the source domain: all model training and, under Setting A defined below, all calibration are confined to this corpus.

PlantDoc, introduced by Singh et al. (2020), was designed to approximate field-based diagnosis more closely. Its images were sourced from internet photographs and real field captures, exhibiting heterogeneous backgrounds, variable sunlight and shadow, multiple overlapping leaves, partial occlusion, motion blur, and a wide range of disease severities. The working PlantDoc archive used here consists of leaf-level crop images organized by disease class. Because the crop filenames alone did not preserve auditable parent-image identifiers, we reconstructed source-image links within each class by indexing the companion source-image archive, resizing every source image and crop to a 32 × 32 RGB thumbnail, flattening these thumbnails into feature vectors, and solving a one-to-one Hungarian assignment on pairwise Euclidean distances. The matched source relative path then served as the parent-image identifier. This procedure recovered source-image identifiers for 2571 of 2572 crops; the single unmatched crop was removed before any split generation. In the retained target set each crop mapped to a unique source image, making parent-image isolation explicit rather than assumed. The resulting collection serves as the target domain for all cross-domain evaluation. This design should be interpreted as a controlled source/field-target leaf-level classification study, not as an end-to-end benchmark of raw-image mobile diagnosis: the localization stage is assumed away, and some full-scene clutter present in original field photographs is reduced by cropping.

Meaningful cross-domain comparison requires a unified category vocabulary. PlantVillage and PlantDoc employ different naming conventions and do not cover identical crop–disease combinations. A category alignment table (**Supplementary Table 1**) was therefore constructed by normalizing all labels to a canonical Crop_Disease format and retaining only those categories for which an unambiguous semantic match existed in both datasets. This intersection yielded *K* = 21 shared categories, hereafter referred to as the known classes. PlantVillage categories absent from PlantDoc were excluded from training so that the classifier’s output space matched the known classes exactly. Conversely, PlantDoc categories with no PlantVillage counterpart were aggregated into an unknown class pool comprising 490 samples across 7 categories. These unknown samples were withheld from all training and calibration procedures and used solely to evaluate the model’s capacity to reject inputs from categories it has never encountered.

Rigorous subset separation is critical for preventing information leakage. The 21-class subset of PlantVillage was split at the image level with stratified random sampling into a training set (70%), a validation set (15%), and a held-out test set (15%). The training set was used exclusively for weight optimization; the validation set served for early stopping, hyperparameter selection, and—under Setting A—for temperature scaling calibration; the test set was reserved for final in-domain reporting and was never consulted during model selection or threshold tuning.

Two experimental settings govern how PlantDoc known samples are allocated. Under Setting A, which simulates the scenario in which no labeled field data are available, the entire matched PlantDoc known set constitutes the cross-domain test set, and temperature scaling as well as threshold selection rely exclusively on the PlantVillage validation split. Under Setting B, 10% of PlantDoc known samples are allocated to a dedicated field calibration set and the remaining 90% to the cross-domain test set, but the split is performed with grouped stratified sampling over reconstructed parent-image identifiers rather than over individual crops. This enforces zero parent-image overlap between calibration and test subsets while approximately preserving class balance. Because our parent-image reconstruction confirmed that every retained PlantDoc crop corresponds to a unique source image, the grouped split yielded the same partition sizes as a simple stratified split; the grouping protocol was retained as a methodological safeguard that would become operative if future dataset versions contained multiple crops per source image, rather than because it altered the allocation in practice. In the representative Setting B split, this procedure yielded 199 calibration crops and 1882 known-class test crops from disjoint source-image groups; Setting A used all 2081 matched known-class crops as the test set. Under both settings the PlantDoc unknown pool remains entirely untouched until final evaluation. All manifests are recorded in a master CSV file that maps every retained crop to its role together with its reconstructed parent-image identifier. Because threshold-based conclusions in Setting B depend directly on the calibration subset, we additionally repeated the grouped 10% calibration split 10 times with seeds 42–51, re-estimated temperature and thresholds on each repeat, and summarize split-sensitive metrics by their mean, standard deviation, and 95% confidence interval.

### 2.2 Primary model, calibration, and selective prediction

The primary case study employed a ResNet-50 (He et al., 2016) pre-trained on ImageNet (Deng et al., 2009) and fine-tuned end-to-end on the PlantVillage training split. The original 1,000-way classification head was replaced by a single fully connected layer mapping the 2,048-dimensional feature representation to 21 logits. This architecture was chosen not for novelty but for its maturity, reproducibility, and well-characterized behavior, consistent with the study’s focus on deployment-oriented reliability assessment rather than incremental accuracy gains.

All input images were resized to 224 × 224 pixels with bilinear interpolation. During training, standard augmentations were applied: random resized cropping with a scale range of [0.8, 1.0], random horizontal and vertical flipping, and color jittering (brightness, contrast, saturation, and hue). During inference, a deterministic resize-plus-center-crop pipeline followed by channel-wise normalization to ImageNet statistics was used. The network was trained by minimizing the cross-entropy loss over the 21 known classes:

(1) $$L_{\text{CE}}=-\frac{1}{N}\sum_{i=1}^{N}\sum_{k=1}^{K}y_{ik}\text{log }{\hat{p}}_{ik}$$

where N denotes the number of training samples, $y_{ik}$ is the one-hot ground-truth indicator, and ${\hat{p}}_{ik}$ is the predicted probability obtained by applying the softmax function to the logit vector $z_{i}$:

(2) $${\hat{p}}_{ik}=\frac{\exp(z_{ik})}{\sum_{j=1}^{K}\exp(z_{ij})}$$

Optimization was conducted with AdamW (Loshchilov and Hutter, 2019) at an initial learning rate of 0.0001, using a cosine annealing schedule over 20 epochs. Weight decay was set to 0.01 and the mini-batch size to 32. Training employed early stopping with patience 5 based on validation accuracy, and the checkpoint with the highest validation accuracy was retained for all downstream evaluation. Experiments were implemented in PyTorch 2.x.

Modern deep networks tend to produce overconfident predictions, and the problem intensifies under domain shift as the model encounters unfamiliar visual patterns yet still generates high logit magnitudes through familiar low-level feature activations. To mitigate miscalibration without altering the trained weights, we applied temperature scaling, a single-parameter *post-hoc* transformation that divides the logit vector by a learned scalar *T* before the softmax operation:

(3) $${\hat{p}}_{ik}^{\left(T\right)}=\frac{\exp(z_{ik}/T)}{\sum_{j=1}^{K}\exp(z_{ij}/T)}$$

When *T* > 1 the distribution is softened, reducing the peak probability and spreading mass more uniformly across classes; when *T* = 1 the original softmax is recovered. For the single-model results, scaling preserves the relative ordering of logits and therefore leaves classification accuracy unchanged. The temperature used for each calibration split was learned by minimizing the negative log-likelihood on that split:

(4) $$T^{*}=\text{arg }\underset{T}{\min}-\sum_{i=1}^{N_{\text{cal}}}\sum_{k=1}^{K}y_{ik}\text{log }{\hat{p}}_{ik}^{\left(T\right)}$$

In practice, this scalar temperature was optimized with L-BFGS initialized at T=1. Under Setting A the calibration set is the PlantVillage validation split ( $T_{A}=1.18$); under the representative Setting B split it is the PlantDoc 10% grouped field split ( $T_{B}=1.54$). Across the 10 grouped Setting B repeats, the mean temperature was 1.5398 (95% CI [1.5346, 1.5450]). The same *post-hoc* calibration logic was applied independently to each backbone. This *post-hoc* calibration setting is part of a broader score-to-probability calibration literature, including multiclass probability calibration and Dirichlet calibration (Zadrozny and Elkan, 2002; Kull et al., 2019). For the DINOv2 ensemble, calibrated classification, calibration, and predictive-confidence results were obtained by applying temperature scaling to the ensemble predictive logits, whereas the aleatoric–epistemic decomposition was retained on the raw member outputs.

Selective prediction augments the classifier with a decision rule that determines, for each input, whether the prediction should be emitted or withheld. We compared four *post-hoc* score functions derived from the calibrated classifier. For maximum softmax probability (MSP):

(5) $$s_{\text{MSP}}(x)=\underset{k}{\max}{\hat{p}}_{k}^{\left(T\right)}(x)$$

For normalized entropy confidence:

(6) $$s_{\text{Ent}}(x)=1-\frac{-\sum_{k=1}^{K}{\hat{p}}_{k}^{\left(T\right)}(x)\text{log}\;{\hat{p}}_{k}^{\left(T\right)}(x)}{\text{log}\;K}$$

For prediction margin:

(7) $$s_{\text{Mar}}(x)={\hat{p}}_{\left(1\right)}^{\left(T\right)}(x)-{\hat{p}}_{\left(2\right)}^{\left(T\right)}(x)$$

where ${\hat{p}}_{\left(1\right)}^{\left(T\right)}(x)$ and ${\hat{p}}_{\left(2\right)}^{\left(T\right)}(x)$ denote the largest and second-largest calibrated probabilities. For the energy-based baseline (Liu et al., 2020):

(8) $$s_{\text{Eng}}\left(x\right)=\text{log}\sum_{k=1}^{K}\text{exp }\left(z_{k}/T\right)\;$$

which equals negative energy; larger values indicate more in-distribution-like samples. Across all four methods, larger scores indicate greater confidence.

For any score function s(x), the selection rule is

(9) $$g_{s}(x)=\{\begin{array}{ll} 1 & \text{if }s(x)\geq \tau \\ 0 & \text{otherwise} \end{array}$$

where τ is the abstention threshold. Samples for which $g_{s}(x)=0$ are routed to human review. Coverage ϕ(τ) is the fraction of test samples that receive an answer:

(10) $$\phi \left(\tau \right)=\frac{1}{N_{test}}\sum_{i=1}^{N_{\text{test}}}g_{s}\left(x_{i}\right)$$

Selective risk $r_{s}(\tau )$ is the error rate among answered samples:

(11) $$r_{s}(\tau )=\frac{\sum_{i=1}^{N_{test}}⊮[{\hat{y}}_{i}\neq y_{i}]\cdot g_{s}(x_{i})}{\sum_{i=1}^{N_{test}}g_{s}(x_{i})}$$

Plotting $r_{s}(\tau )$ against ϕ(τ) as τ varies produces the coverage–risk curve. Two threshold selection strategies, both calibrated exclusively on the calibration set, are reported: a fixed-coverage strategy in which τ is set to achieve target coverage levels (90%, 80%, 70%, 60%, 50%), and a fixed-risk strategy in which τ is set so that calibration-set selective risk does not exceed a target level (5%, 10%, 15%). Thresholds are then applied to the test set without modification. The same threshold is additionally applied to the unknown-class pool to compute the unknown rejection rate—the proportion of unknown-class samples whose score falls below τ—which quantifies the model’s ability to avoid issuing confident yet necessarily incorrect diagnoses for categories absent from training. The complementary known-class rejection rate captures the cost of excessive conservatism.

### 2.3 Domain shift quantification and attention analysis

To move beyond aggregate accuracy comparisons and understand the structural basis of the domain gap, we computed a suite of image-level visual statistics on PlantVillage and PlantDoc images from the shared known classes. For each image, 17 low-level descriptors were extracted, covering color distribution (mean and variance in HSV saturation and value channels), spatial frequency content (Laplacian variance, Sobel edge density, border-region edge density), texture (grayscale entropy and local binary pattern entropy), and a foreground-occupancy proxy based on saturation and edge activity. For each descriptor, image-level values were first averaged within each class and domain, and the paired two-domain difference across classes was quantified using a Wilcoxon signed-rank test and Cohen’s *d* effect size. Bonferroni correction was then used when interpreting the family of descriptor tests. This analysis provides an interpretable, model-free characterization of how the two corpora differ at the pixel level, complementing the model-dependent reliability metrics reported elsewhere.

To examine whether the fine-tuned ResNet-50 attends to diagnostically relevant regions or to spurious background cues, we applied Gradient-weighted Class Activation Mapping (GradCAM) (Selvaraju et al., 2017) to matched source–target image pairs. For each pair, a PlantVillage image and a PlantDoc image belonging to the same disease category were selected and passed through the trained model. Grad-CAM heatmaps were generated from the final convolutional layer and overlaid on the input images. The analysis focused on cases where the model produced a high-confidence prediction on both images—correct on the source image but incorrect on the target image—to illustrate how attention shifts between domains. To quantify the spatial distribution of attention, the fraction of Grad-CAM activation mass falling within a border strip (the outer 20% of image width and height) was computed as a border activation ratio. Higher border ratios on target images indicate that the model’s attention has drifted from central lesion regions toward peripheral background cues.

### 2.4 Target-aware mitigation baselines

Three lightweight adaptation strategies were evaluated as target-aware mitigation baselines, all applied to the fine-tuned ResNet-50 representation. Each strategy uses the Setting B target calibration subset only for unlabeled target statistics or domain alignment during adaptation; target labels are used only later for the standard temperature and threshold estimation steps defined by the evaluation protocol.

For adaptive batch normalization (AdaBN), following Li et al. (2017), the batch normalization running statistics (mean and variance) of the fine-tuned ResNet-50 were replaced with statistics computed from a forward pass over the Setting B calibration subset of PlantDoc images. All other model parameters, including the classification head, remained unchanged. This procedure adapts the model’s internal feature normalization to target-domain activation scales at negligible computational cost.

For feature moment matching, penultimate-layer ResNet-50 features were used to construct a diagonal moment-matching baseline. The PlantVillage training features were affine-transformed so that their channel-wise mean and variance matched the corresponding statistics estimated from the Setting B PlantDoc calibration subset. Specifically, given source features h, the aligned features were computed as $\tilde{h}=\sigma_{t}(h-\mu_{s})/\sigma_{s}+\mu_{t}$, where $\mu_{s},\sigma_{s}$ are source-domain channel statistics and $\mu_{t},\sigma_{t}$ are target-domain channel statistics estimated from the calibration subset. A linear classifier was then trained on the aligned source features and evaluated on PlantDoc features.

For the domain-adversarial neural network (DANN) comparison, following Ganin et al. (2016), we implemented a feature-space DANN-style baseline on top of fixed penultimate ResNet-50 features. A small bottleneck classifier was trained with source-label supervision on PlantVillage features, while a gradient reversal branch fed a binary domain discriminator trained to distinguish PlantVillage from unlabeled PlantDoc calibration features. This adversarial alignment strategy is included as a negative-result control: it represents a stronger adaptation method that, in principle, should encourage domain-invariant features but may also disrupt task-relevant discriminative structure under severe shift.

All three baselines were evaluated under the same repeated grouped Setting B protocol described in Section 2.1, with 10 repeats.

### 2.5 Backbone and ensemble baselines

To test whether the reliability conclusions drawn from the fine-tuned ResNet-50 generalize across representation families, we conducted a standardized comparison using five backbones spanning three pre-training paradigms and multiple architecture families: (i) ResNet-50 with ImageNet-supervised pre-training (CNN family), (ii) ViT-S/16 with ImageNet-supervised pre-training (Dosovitskiy et al., 2021) (supervised transformer family), (iii) DINOv2-S/14 with self-supervised pre-training (Oquab et al., 2024) (self-supervised transformer family), (iv) CLIP ViT-B/16 (Radford et al., 2021) (vision–language pre-training, transformer), and (v) CLIP ResNet-50 (Radford et al., 2021) (vision–language pre-training, CNN). To ensure that performance differences reflect representation quality rather than optimization or capacity confounds, all five backbones were evaluated under a unified frozen-encoder protocol. Each encoder was loaded with its publicly available pre-trained weights and kept entirely frozen; images were resized to the encoder’s native resolution and passed through the frozen encoder to extract a fixed-dimensional feature vector. A single linear classification head was then trained on the PlantVillage training split using the same loss, optimizer, and early stopping criteria described in Section 2.2. The same PlantDoc grouped-split protocol, calibration procedure, and uncertainty evaluation logic were applied identically to all backbones. This design isolates the effect of the pre-trained representation while holding the downstream pipeline constant. Because the frozen-representation protocol is deliberately more constrained than full end-to-end fine-tuning, the comparison characterizes how much of the reliability gap can be attributed to representation quality alone, without confounding it with differences in fine-tuning depth or optimization landscape. This asymmetry between the primary fine-tuned case study and the frozen-feature backbone comparison is intentional: the former measures deployment risk under a realistic training protocol, while the latter isolates representation quality as a single variable.

Two ensemble baselines were constructed to assess whether model-level aggregation provides additional calibration or uncertainty benefits beyond single-model scoring.

For the warm-start end-to-end ResNet-50 ensemble, we constructed a five-member baseline from the fine-tuned ResNet-50 to provide a more credible end-to-end ensemble than a shared-feature linear probe ensemble can offer. The root fine-tuned checkpoint (Section 2.2) was retained as ensemble member 1. Four additional members were created by warm-starting from this root checkpoint and continuing end-to-end fine-tuning on bootstrap subsamples (sampling with replacement) of the PlantVillage training set, each with a different random seed. This design introduces diversity through both data resampling and optimization trajectory divergence, while remaining computationally practical. It should be noted that this is not a fully independent cold-start deep ensemble in the sense of Lakshminarayanan et al. (2017), because all members share the same pre-trained initialization; the resulting diversity is therefore a lower bound on what a true deep ensemble would provide.

As a supplementary limited-diversity baseline, a lightweight DINOv2 linear-probe ensemble was constructed on top of the frozen DINOv2-S/14 backbone, which achieved the strongest single-model cross-domain performance. Five linear classification heads were trained independently on the same frozen features, differing only in random initialization seed. Because all members share identical visual representations and differ only in the linear classifier, this ensemble captures sensitivity to classifier initialization—a narrow form of uncertainty that does not reflect disagreement about visual features. Its epistemic uncertainty estimates should therefore be interpreted as a practical disagreement diagnostic rather than a comprehensive model-uncertainty measure.

For the ensemble uncertainty decomposition, the predictive distribution was obtained by averaging the softmax outputs of the *M* = 5 members:

(12) $${\bar{p}}_{k}\left(x\right)=\frac{1}{M}\sum_{m=1}^{M}p_{k}^{\left(m\right)}\left(x\right)$$

Three uncertainty measures were derived from this averaged distribution. Predictive entropy captures total uncertainty:

(13) $$H[\bar{p}]=-\sum_{k=1}^{K}{\bar{p}}_{k}\text{log }{\bar{p}}_{k}$$

Mean member entropy serves as an aleatoric proxy:

(14) $$E[H[p^{\left(m\right)}]]=\frac{1}{M}\sum_{m=1}^{M}\left(-\sum_{k=1}^{K}p_{k}^{\left(m\right)}\text{log }p_{k}^{\left(m\right)}\right)$$

Mutual information between the prediction and the model index provides an epistemic proxy:

(15) $$\text{MI}=H[\bar{p}]-E[H[p^{\left(m\right)}]]$$

Both ensembles were evaluated under the same repeated grouped Setting B protocol with 10 repeats.

### 2.6 Evaluation metrics and statistical analysis

The evaluation framework spans three complementary dimensions. Classification performance is measured by top-1 accuracy and macro-averaged F1 score. Calibration quality is assessed through reliability diagrams (DeGroot and Fienberg, 1983), expected calibration error (ECE) (Naeini et al., 2015), and the Brier score (Brier, 1950). The reliability diagram partitions the predicted confidence range into *M* = 15 equal-width bins and plots mean predicted confidence against empirical accuracy. Fifteen bins were retained for consistency with the original reliability diagrams and because this bin count provides an interpretable, standard calibration summary that remains stable across the repeated grouped splits used in this study; the Brier score is reported alongside ECE as a strictly proper scoring rule that does not depend on bin discretization. ECE summarizes the reliability diagram into a single scalar:

(16) $$\text{ECE}=\sum_{m=1}^{M}\frac{\left|B_{m}\right|}{N}\left|\text{acc}\left(B_{m}\right)-\text{conf}\left(B_{m}\right)\right|$$

where $\left|B_{m}\right|$ is the number of samples in bin m, $\text{acc}(B_{m})$ is the empirical accuracy within the bin, and $\text{conf}(B_{m})$ is the mean predicted confidence. The Brier score offers a strictly proper assessment that jointly penalizes miscalibration and lack of discrimination:

(17) $$\text{Brier}=\frac{1}{N}\sum_{i=1}^{N}\sum_{k=1}^{K}{\left(y_{ik}-{\hat{p}}_{ik}\right)}^{2}$$

Together, Equations 1–17 define the training objective, calibration transform, confidence scores, selective-prediction rules, ensemble uncertainty quantities, and calibration metrics used throughout the evaluation. For selective prediction, we report selective risk at fixed target coverage levels and achieved coverage under fixed target-risk constraints, where thresholds are selected on calibration data and transferred to the test set. To supplement operating-point results with threshold-independent open-set metrics, we additionally compute AUROC, average precision with unknown samples as the positive class (AUPR-Unknown), and the false positive rate at 95% true positive rate (FPR95). AUROC is retained as a widely used threshold-independent ranking summary, but because AUROC can be insensitive to class-imbalance effects—the unknown pool (490 samples) is smaller than the known test set—AUPR-Unknown is included in the synthesis table and FPR95 is reported in the score-comparison tables to provide complementary perspectives on separation quality and worst-case false-alarm rates. All metrics are computed on both the PlantVillage test set (in-domain reference) and the PlantDoc test set (cross-domain evaluation), reported separately for Settings A and B. Setting A and the representative grouped Setting B split are reported as point estimates, while Setting B split-sensitive quantities are additionally summarized over 10 grouped repeats using mean values, standard deviations, and 95% confidence intervals.

For the backbone comparison and ensemble analyses, between-backbone differences in accuracy, macro-F1, calibrated ECE, and calibrated Brier score were evaluated using paired Wilcoxon signed-rank tests across the 10 repeated splits, with each split serving as a paired observation. This non-parametric test was chosen because the number of repeats is modest and normality of the difference distribution cannot be assumed. The same pairing structure was used to compare uncertainty-score performance across backbone families.

## 3 Results

The empirical findings are organized into six modules that collectively characterize the deployment risk of controlled-domain classifiers applied to field conditions. Module 1 documents the severity of the reliability gap in the primary fine-tuned ResNet-50 case study, covering accuracy collapse, miscalibration, selective prediction failure, and open-set rejection. Module 2 explains why the gap is severe through quantitative shift statistics and Grad-CAM attention analysis. Module 3 benchmarks simple target-aware mitigations. Module 4 examines whether stronger representations narrow the gap. Module 5 evaluates ensemble baselines. Module 6 provides a concise cross-method synthesis.

### 3.1 Severity of the reliability gap

This subsection documents the simultaneous failure of accuracy, calibration, selective prediction, and open-set rejection in the primary fine-tuned ResNet-50 case study.

**Table 1** reports classification performance. On PlantVillage the model achieved 99.73% accuracy, consistent with prior work. When transferred to PlantDoc, accuracy fell to 32.05% under Setting A— a 67.7-percentage-point drop—while mean predicted confidence remained at 79.76%. Across the 10 grouped Setting B repeats, raw accuracy averaged 32.10% (95% CI [31.88%, 32.32%]) and macro-F1 averaged 0.2859 (95% CI [0.2836, 0.2881]), confirming the result is robust to split variation. The simultaneous occurrence of near-random accuracy and high confidence is the defining feature of the reliability gap.

**Table 1:** Classification performance of the fine-tuned ResNet-50 on in-domain (PlantVillage) and cross-domain (PlantDoc) test sets.

Dataset	Accuracy (%)	Macro F1	Mean $p_{\text{max }}$
PlantVillage Test (In-Domain)	99.73	0.9959	0.9982
PlantDoc Known Test - Setting A	32.05	0.2853	0.7976
PlantDoc Known Test - Setting B	32.04	0.2836	0.7965

**Table 2**, **Figure 1** present calibration results. *Post-hoc* temperature scaling provided measurable but insufficient improvements. Under Setting A (source-only calibration), ECE decreased from 0.4771 to 0.4400 (7.8% relative). Under Setting B (small grouped field calibration set), the reduction was larger: ECE dropped from 0.4761 to 0.3651 (23.3% relative), and Brier score fell from 1.1124 to 1.0083. The repeated grouped-split analysis confirms stability: mean calibrated ECE was 0.3645 (95% CI [0.3611, 0.3679]) and calibrated Brier 1.0051 (95% CI [1.0014, 1.0089]) across 10 repeats (**Table 3**). Improved calibration, however, should not be mistaken for sufficient reliability: a residual ECE above 0.36 indicates that predicted confidence still substantially exceeds empirical accuracy.

**Table 2:** Calibration metrics before and after temperature scaling (fine-tuned ResNet-50).

Dataset	Calibration	ECE	Brier Score	Mean $p_{\text{max }}$
PlantVillage Test	Before (*T* = 1)	0.0018	0.0051	0.9982
PlantDoc Known (Setting A)	Before (*T* = 1)	0.4771	1.1104	0.7976
PlantDoc Known (Setting A)	After (*T* = 1.18)	0.4400	1.0728	0.7605
PlantDoc Known (Setting B)	Before (*T* = 1)	0.4761	1.1124	0.7965
PlantDoc Known (Setting B)	After (*T* = 1.54)	0.3651	1.0083	0.6855

**Figure 1:**
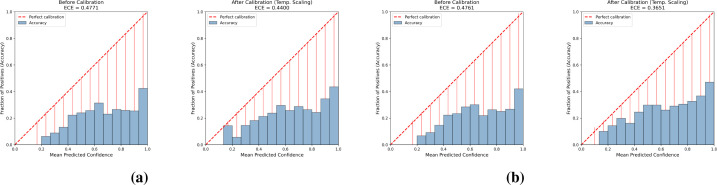
Reliability diagrams showing predicted confidence versus empirical accuracy before and after temperature scaling. **(a)** PlantDoc known-class evaluation under Setting A, where temperature scaling is calibrated on the PlantVillage validation split (source-only calibration; T = 1.18). **(b)** PlantDoc known-class evaluation under Setting B, where temperature scaling is calibrated on the 10% grouped PlantDoc field calibration subset (T = 1.54). In each panel, the left plot shows the uncalibrated model (T = 1) and the right plot shows the temperature-scaled model.

**Table 3:** Robustness of setting B across 10 parent-image-aware calibration/test splits (fine-tuned ResNet-50).

Quantity	Mean	95% CI
Temperature *T*_*B*_	1.5398	[1.5346, 1.5450]
Accuracy (raw)	0.3210	[0.3188, 0.3232]
ECE (raw)	0.4766	[0.4741, 0.4790]
ECE (calibrated)	0.3645	[0.3611, 0.3679]
Brier score (calibrated)	1.0051	[1.0014, 1.0089]
Coverage at nominal 80% (calibrated)	0.8088	[0.7906, 0.8270]
Selective risk at nominal 80% (calibrated)	0.6430	[0.6399, 0.6461]
Coverage under 5–15% target risk (calibrated)	0.0029	[0.0006, 0.0052]
Actual risk under 5–15% target risk (calibrated)	0.2661	[0.0910, 0.4412]

**Figure 2:**
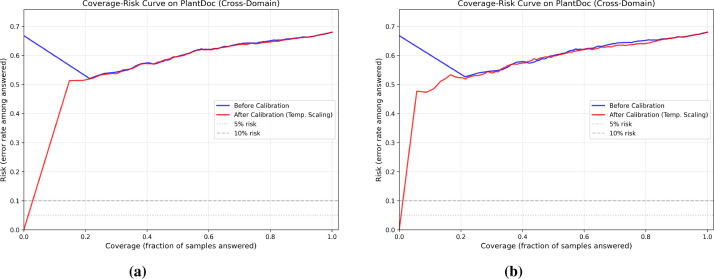
Coverage–risk curves showing the trade-off between the fraction of samples answered and the error rate among answered samples. **(a)** Setting A, with thresholds selected using the PlantVillage validation split and transferred to the PlantDoc known set. **(b)** Setting B, with thresholds selected using the grouped PlantDoc field calibration subset and applied to the held-out PlantDoc known test set. Blue curves denote before calibration and red curves denote after temperature scaling; dashed gray lines mark 5% and 10% risk levels.

**Figure 2** shows the coverage–risk trade-off. Under Setting A, source-domain thresholds collapsed effective coverage: at nominal 80%, only 5.1% of samples were answered. Under Setting B, threshold transfer was more stable but risk remained extremely high. Across 10 grouped repeats, calibrated selective risk averaged 0.6430 (95% CI [0.6399, 0.6461]) at nominal 80% coverage and 0.5939 (95% CI [0.5841, 0.6037]) at nominal 50% coverage (**Table 3**). Fixed-risk thresholding was not practically usable: targeting 5–15% maximum risk produced mean coverage of only 0.0029 with mean actual risk of 0.2661. Threshold transfer should therefore be interpreted as a property of the evaluation procedure, not as evidence of deployment-grade reliability.

Softmax confidence provides limited discriminative power for open-set detection. At a practical MSP threshold of = 0.70, the model rejected 45.71% of unknown-class samples while simultaneously rejecting 37.77% of known-class samples—a gap of only 7.94 percentage points. At the permissive threshold *τ* = 0.30, only 4.29% of unknown-class samples were rejected, demonstrating that the model frequently assigns high confidence to inputs from categories it has never seen.

Replacing MSP with entropy, margin, or energy scoring yielded modest improvements (**Table 4**). Entropy achieved the highest mean unknown-detection AUROC (0.6101; 95% CI [0.6088, 0.6113]), while energy achieved the lowest mean FPR95 (0.8282; 95% CI [0.8267, 0.8297]) and the lowest mean selective risk at nominal 50% coverage (0.5851; 95% CI [0.5782, 0.5919]). At nominal 80% coverage, however, all four scoring methods remained clustered between 0.6430 and 0.6545 mean risk. The broader conclusion therefore does not depend on MSP alone: the deployment-grade reliability gap persists across all standard *post-hoc* scoring functions within the ResNet-50 case study.

**Table 4:** Repeated grouped setting B comparison of score-based uncertainty baselines (fine-tuned ResNet-50).

Score	AUROC	FPR95	Risk @ nominal 80%	Risk @ nominal 50%	Coverage @ 10% target risk
MSP	0.5832 [0.5816, 0.5847]	0.8623 [0.8608, 0.8638]	0.6430 [0.6399, 0.6461]	0.5939 [0.5841, 0.6037]	0.0029 [0.0006, 0.0052]
Entropy	0.6101 [0.6088, 0.6113]	0.8489 [0.8474, 0.8504]	0.6456 [0.6416, 0.6496]	0.5929 [0.5855, 0.6003]	0.0032 [0.0007, 0.0057]
Margin	0.5656 [0.5638, 0.5674]	0.8633 [0.8620, 0.8646]	0.6545 [0.6523, 0.6567]	0.5960 [0.5883, 0.6037]	0.0026 [0.0003, 0.0048]
Energy	0.6070 [0.6055, 0.6085]	0.8282 [0.8267, 0.8297]	0.6442 [0.6401, 0.6483]	0.5851 [0.5782, 0.5919]	0.0040 [0.0019, 0.0060]

Values are mean [95% CI].

### 3.2 Why the gap is severe: shift quantification and attention analysis

**Figure 3:**
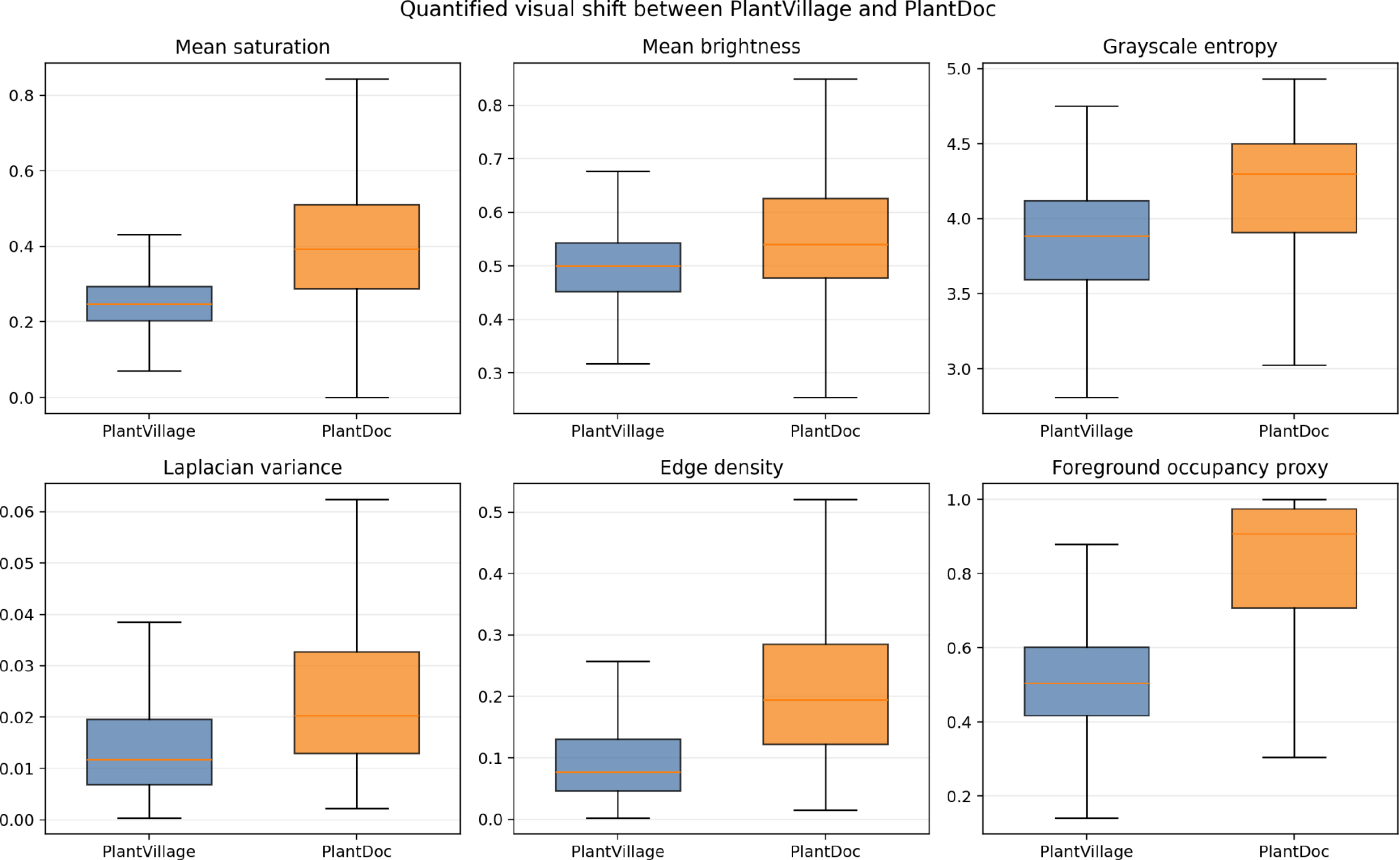
Visual shift quantification overview. Distributions of key image-level statistics for PlantVillage (source) and PlantDoc (target) corpora, illustrating the large-effect-size differences in saturation, edge density, foreground-occupancy proxy, and textural roughness.

The severity of the cross-domain reliability gap documented above is not merely a calibration artifact; it has a structural basis in measurable visual distribution differences between the two corpora. **Table 5** summarizes the image-level shift analysis. The six most discriminative metrics, all with large effect sizes, were: mean HSV saturation (*d* = 3.90), border edge density (*d* = 3.33), overall edge density (*d* = 2.60), foreground-occupancy proxy (*d* = 2.48), color variance (*d* = 2.30), and Laplacian variance (*d* = 1.63). All six differences remained statistically significant under Bonferroni correction at *α* = 0.05 (raw paired Wilcoxon *p* ≤ 1.81 × 10^−5^). PlantDoc images exhibit substantially higher saturation variability, denser edge structure (especially near image borders), higher foreground-occupancy proxy values, and higher textural roughness than PlantVillage images. These differences are consistent with the shift from uniform single-leaf photographs to heterogeneous field-sourced images containing cluttered backgrounds, variable illumination, and multiple overlapping leaves. These cross-domain distributional shifts are visualized in **Figure 3**.

**Table 5:** Quantitative shift characterization: top six image-level visual statistics distinguishing PlantVillage from PlantDoc (paired Wilcoxon signed-rank tests on class means; Bonferroni-corrected interpretation).

Metric	Cohen’s *d*	Δ (PDoc − PVil)	*p*-value
Mean saturation (HSV)	3.90	+0.159	9.54 × 10^−7^
Border edge density	3.33	+0.130	9.54 × 10^−7^
Edge density (Sobel)	2.60	+0.113	9.54 × 10^−7^
Foreground occupancy proxy	2.48	+0.276	9.54 × 10^−7^
Color variance	2.30	+0.023	6.68 × 10^−6^
Laplacian variance	1.63	+0.010	1.81 × 10^−5^

**Figure 4:**
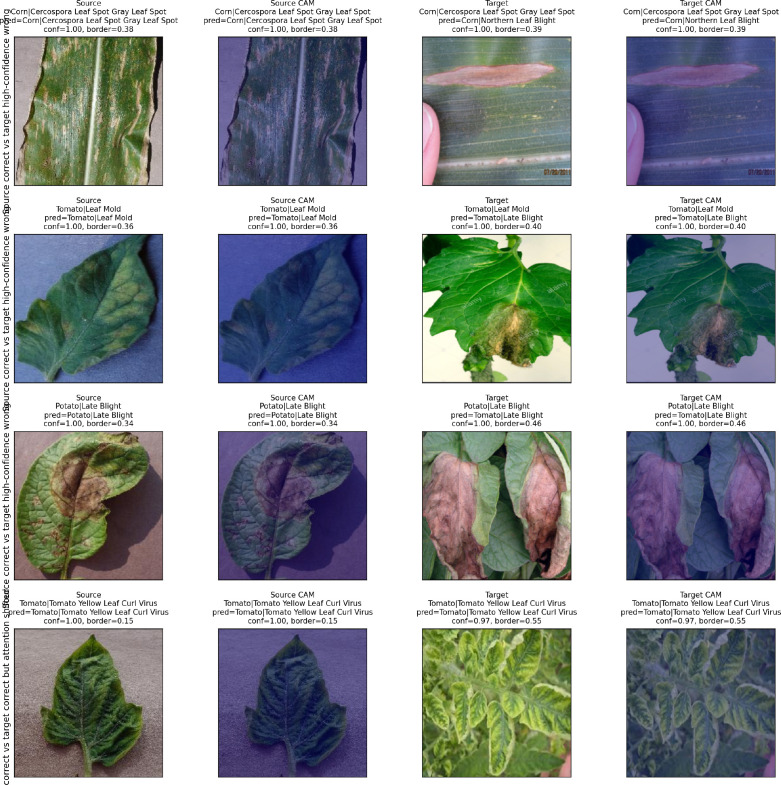
Grad-CAM visualizations on matched source (PlantVillage) and target (PlantDoc) pairs. In three of four cases, the model produces a high-confidence incorrect prediction on the target image while shifting attention from central lesion regions to background-dominated areas. Border activation ratios quantify the fraction of Grad-CAM mass in the outer 20% of the image.

Grad-CAM visualizations on matched source–target pairs provide complementary evidence at the model level. **Figure 4** shows four matched pairs from disease categories in which the model produces high-confidence predictions on both source and target images but is correct only on the source. In three of the four cases—Corn Gray Leaf Spot misclassified as Northern Leaf Blight (confidence 0.9999998), Tomato Leaf Mold misclassified as Late Blight (confidence 0.9999987), and Potato Late Blight misclassified as Tomato Late Blight (confidence 0.9999975)—the model’s attention shifted from central lesion regions in the source image to background-dominated or border-adjacent areas in the target image. In the fourth case, Tomato Yellow Leaf Curl Virus, the model predicted correctly on both images but the border activation ratio increased from 0.15 (source) to 0.55 (target), indicating that even correct predictions relied more heavily on peripheral cues in the field image. Together, the shift quantification and Grad-CAM evidence establish that the reliability gap is driven by identifiable visual distribution differences that cause the model to attend to spurious rather than diagnostically relevant features under field conditions.

### 3.3 Simple target-aware mitigations

Three lightweight adaptation baselines were evaluated to test whether simple target-aware corrections can substantially narrow the reliability gap. All three were applied to the fine-tuned ResNet-50 and evaluated under the same repeated grouped Setting B protocol.

Replacing the batch normalization running statistics with target-domain statistics improved accuracy from 0.321 to 0.343 (95% CI [0.341, 0.346]) and reduced calibrated ECE from 0.365 to 0.288 (95% CI [0.282, 0.294]). The calibrated Brier score decreased from 1.005 to 0.911. Unknown-detection AUROC using energy scoring improved marginally from 0.610 to 0.611, and selective risk at nominal 80% coverage fell from 0.643 to 0.623. AdaBN thus provides a modest, low-cost improvement, but leaves all deployment-relevant metrics far from adequate.

**Figure 5:**
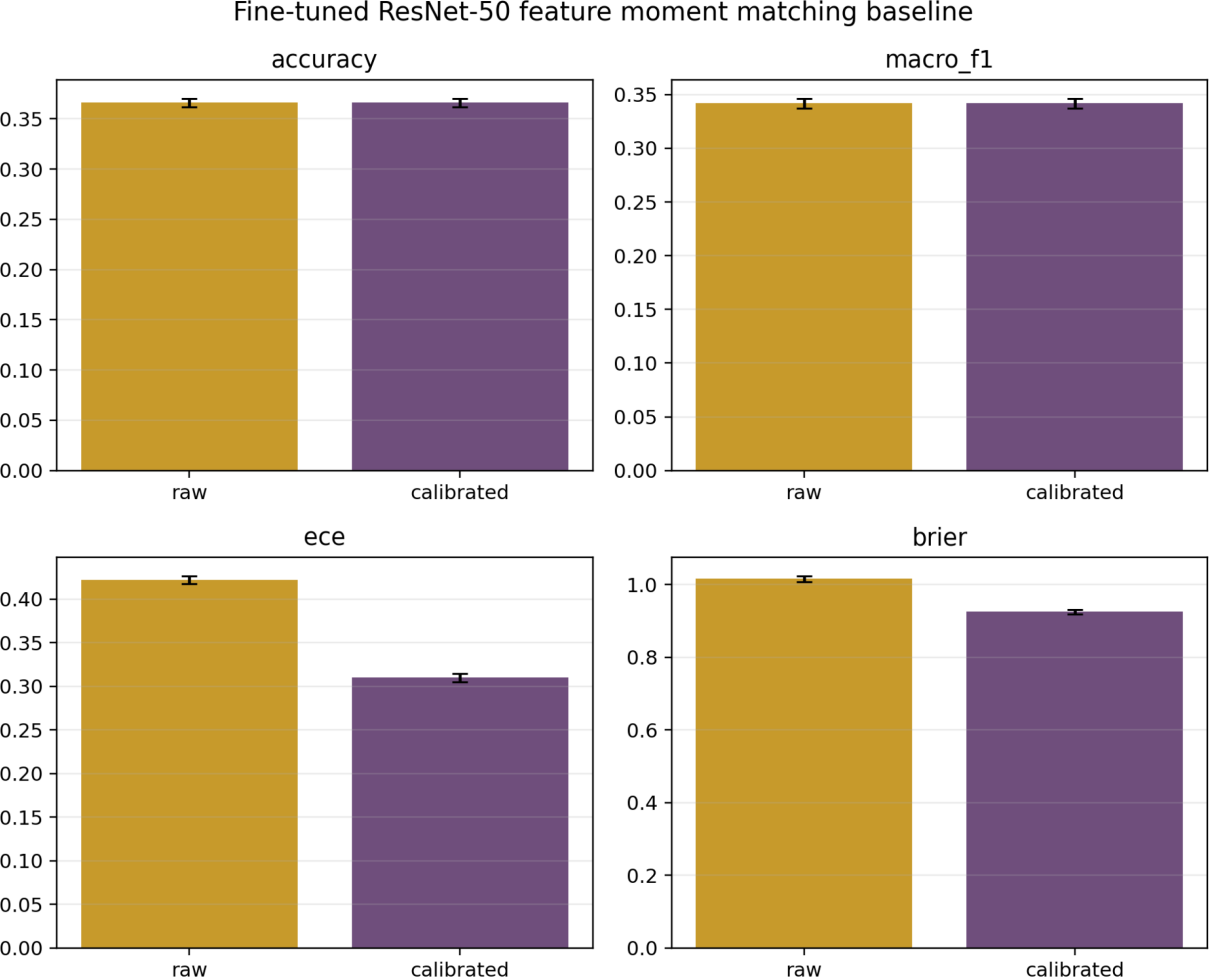
Key classification and calibration metrics for the feature moment-matching baseline applied to the fine-tuned ResNet-50 under repeated grouped Setting B evaluation. Bars compare raw and temperature-scaled outputs; error bars indicate standard deviations across the 10 calibration/test splits. Temperature scaling improves ECE and Brier score, while accuracy and macro-F1 remain unchanged because post-hoc scaling does not change predicted labels.

Aligning source feature statistics to target-domain moments achieved the strongest simple adaptation results. Accuracy improved to 0.366 (95% CI [0.363, 0.369]), ECE decreased to 0.310 (95% CI [0.306, 0.313]), and Brier score fell to 0.925. Notably, unknown-detection AUROC improved to 0.648, and selective risk at nominal 80% coverage dropped to 0.594, the lowest among the adaptation baselines. Moment matching thus demonstrates that target-aware feature alignment can yield meaningful operational gains beyond what pure *post-hoc* calibration offers. **Figure 5** summarizes the corresponding raw and temperature-scaled classification and calibration metrics for the feature moment-matching baseline.

In contrast, domain-adversarial training degraded performance. Accuracy fell to 0.252 (95% CI [0.227, 0.277])—below the unadapted baseline—and variance across repeats was high (ECE: 0.245, 95% CI [0.155, 0.335]). Unknown-detection AUROC was 0.577, and selective risk at nominal 80% coverage rose to 0.717. This negative result suggests that adversarial feature alignment, while effective in other settings, can disrupt task-relevant discriminative structure under the severe and heterogeneous shift present in this benchmark.

The overall pattern is clear: simple target-aware corrections help, and moment matching produces the strongest gains among them. But even the best simple adaptation baseline leaves accuracy below 37%, selective risk at nominal 80% coverage near 0.59, and unknown-detection AUROC substantially below the strongest frozen-backbone baseline (Section 3.4). Standard lightweight adaptation reduces but does not eliminate the reliability gap.

### 3.4 Effect of stronger representations

A natural question is whether the reliability failures are peculiar to a single convolutional architecture or whether they persist across fundamentally different representation families. To address this, we evaluated five backbones under the standardized frozen-representation protocol described in Section 2.5.

**Table 6** reports classification performance under the frozen-representation protocol. All five backbones achieved near-ceiling in-domain accuracy on PlantVillage (97–99%), confirming that the frozen-encoder protocol is sufficient for the source domain. Cross-domain performance, however, differed markedly. Under the repeated grouped Setting B evaluation, DINOv2-S/14 achieved the highest mean accuracy (43.19%; 95% CI [43.02%, 43.37%]), followed by ViTS/16 (41.88%), CLIP ViT-B/16 (41.38%), CLIP ResNet-50 (29.15%), and frozen ResNet-50 (28.02%). The self-supervised and vision–language transformer backbones thus provided a roughly 13–15-percentage-point advantage over the CNN baselines.

**Table 6:** Classification performance across backbone families under the standardized frozen-representation protocol (single split).

Backbone	Pretraining	PV test Acc. (%)	PV test F1	Doc setting A acc. (%)	Doc setting B acc. (%)	Doc setting B F1
ResNet-50	ImageNet supervised	98.03	0.9775	28.06	27.74	0.2350
ViT-S/16	ImageNet supervised	98.65	0.9836	42.05	41.39	0.3819
DINOv2-S/14	Self-supervised	98.48	0.9815	43.06	42.56	0.3899
CLIP ViT-B/16	Vision–language	98.28	0.9795	41.52	41.18	0.3763
CLIP ResNet-50	Vision–language	98.10	0.9781	29.17	29.06	0.2559

Calibration quality also improved with stronger representations (**Table 7**): calibrated ECE was lowest for CLIP ViT-B/16 (0.268; 95% CI [0.265, 0.270]), followed by ViT-S/16 (0.303) and DINOv2-S/14 (0.309), all substantially below the frozen ResNet-50 value (0.331). Calibrated Brier score was lowest for DINOv2-S/14 [0.830; 95% CI (0.827, 0.834)]. Paired Wilcoxon signed-rank tests across the 10 grouped repeats confirmed statistically significant pairwise differences in accuracy and macro-F1 for all backbone pairs (**p*_<_*0.005). Calibration differences were also significant for most pairwise comparisons, with the closest exceptions being calibrated ECE between ResNet-50 and CLIP ResNet-50 (= 0.027) and calibrated Brier score between DINOv2-S/14 and CLIP ViT-B/16 (*p* = 0.0098). CLIP ResNet-50, despite its vision–language pre-training, performed comparably to frozen ImageNet-supervised ResNet-50, indicating that not all modern pre-training paradigms automatically improve cross-domain reliability when combined with a CNN architecture.

**Table 7:** Repeated grouped setting B summary across backbone families (frozen-representation protocol).

Backbone	Accuracy	Macro-F1	Calibrated ECE	Calibrated brier
ResNet-50	0.2802 [0.2770, 0.2834]	0.2381 [0.2348, 0.2415]	0.3310 [0.3272, 0.3349]	1.0039 [0.9996, 1.0083]
ViT-S/16	0.4188 [0.4172, 0.4204]	0.3876 [0.3858, 0.3893]	0.3025 [0.2998, 0.3053]	0.8603 [0.8573, 0.8634]
DINOv2-S/14	0.4319 [0.4302, 0.4337]	0.3964 [0.3944, 0.3984]	0.3093 [0.3068, 0.3118]	0.8302 [0.8267, 0.8337]
CLIP ViT-B/16	0.4138 [0.4127, 0.4149]	0.3786 [0.3774, 0.3797]	0.2679 [0.2654, 0.2705]	0.8357 [0.8330, 0.8383]
CLIP ResNet-50	0.2915 [0.2899, 0.2931]	0.2585 [0.2563, 0.2608]	0.3373 [0.3341, 0.3404]	0.9806 [0.9782, 0.9831]

Mean [95% CI] over 10 repeats.

Stronger representations also improved uncertainty-based scoring. **Table 8** compares all five backbones using energy scoring, which yielded the highest unknown-detection AUROC consistently across backbones. DINOv2-S/14 achieved the highest AUROC [0.7637; 95% CI (0.7628, 0.7647)], followed by ViT-S/16 (0.7517). CLIP ViT-B/16 achieved 0.6282—competitive but substantially below the self-supervised baseline—while both CNN architectures (frozen ResNet-50 and CLIP ResNet-50) achieved AUROC near 0.61–0.68. Within this energy-scoring comparison, DINOv2-S/14 also achieved the lowest selective risk at nominal 80% coverage (0.5197), whereas CLIP ViT-B/16 had the lowest risk at nominal 50% coverage (0.4595). The corresponding risks for frozen ResNet-50 were 0.6911 at 80% coverage and 0.6356 at 50% coverage. The overall pattern is consistent: representation choice materially affects uncertainty-based performance, yet even the most favorable backbone–score pairing left selective risk at nominal 80% coverage above 0.51 and FPR95 above 0.81, indicating heavy overlap between the known and unknown score distributions.

**Table 8:** Cross-backbone comparison under energy scoring in repeated grouped setting B.

Backbone	AUROC unknown	FPR95	Risk @ 80%	Risk @ 50%	Coverage @ 10% risk
ResNet-50	0.6760 [0.6753, 0.6767]	0.7366 [0.7352, 0.7380]	0.6911 [0.6869, 0.6953]	0.6356 [0.6303, 0.6409]	0.0055 [0.0016, 0.0094]
ViT-S/16	0.7517 [0.7507, 0.7527]	0.7741 [0.7721, 0.7761]	0.5508 [0.5453, 0.5564]	0.4978 [0.4952, 0.5003]	0.0097 [0.0014, 0.0179]
DINOv2-S/14	0.7637 [0.7628, 0.7647]	0.8138 [0.8125, 0.8151]	0.5197 [0.5145, 0.5249]	0.4736 [0.4691, 0.4782]	0.0100 [0.0037, 0.0164]
CLIP ViT-B/16	0.6282 [0.6271, 0.6293]	0.8555 [0.8541, 0.8568]	0.5324 [0.5261, 0.5387]	0.4595 [0.4542, 0.4648]	0.0207 [0.0047, 0.0368]
CLIP ResNet-50	0.6117 [0.6107, 0.6127]	0.8802 [0.8787, 0.8818]	0.6667 [0.6629, 0.6705]	0.6023 [0.5972, 0.6075]	0.0076 [0.0030, 0.0122]

Mean [95% CI] over 10 repeats. Energy scoring is shown because it yielded the highest unknown-detection AUROC across all five backbones.

**Figure 6:**
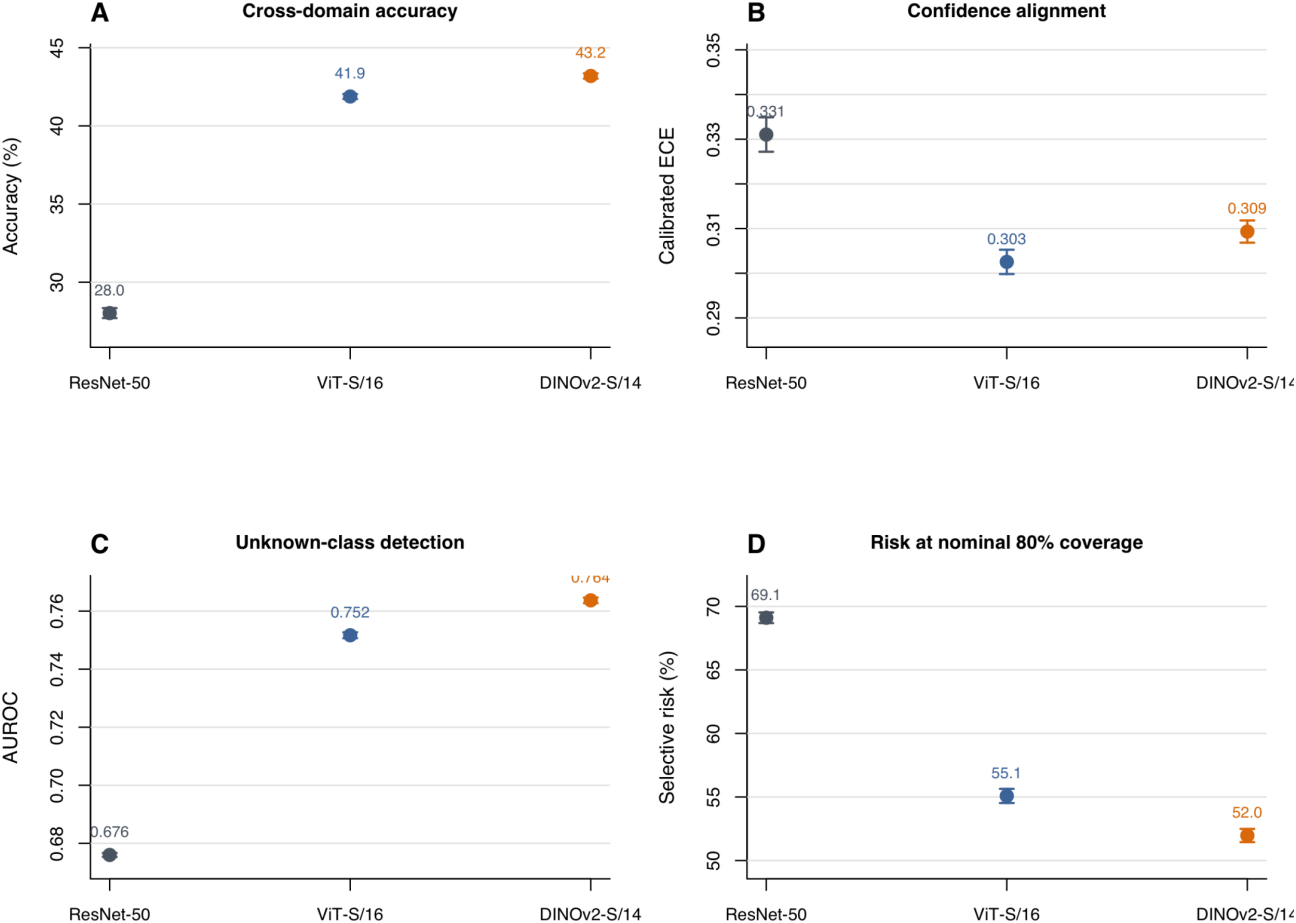
Representative summary of backbone sensitivity under repeated grouped Setting B evaluation for the three frozen-feature backbones shown in the figure: ResNet-50, ViT-S/16, and DINOv2-S/14. Panels report mean values with 95% confidence intervals over 10 repeats for **(A)** cross-domain accuracy, **(B)** calibrated expected calibration error, **(C)** unknown-detection AUROC using energy scoring, and **(D)** selective risk at nominal 80% coverage using energy scoring. The full five-backbone numerical comparison and pairwise statistical conclusions, including CLIP ViT-B/16 and CLIP ResNet-50, are reported in **Tables 6**–**8** and the accompanying Results text.

**Figure 6** consolidates these trends across four deployment-relevant axes. Transformer-based representations improved all four dimensions relative to ResNet-50, with DINOv2-S/14 providing the strongest overall balance: the highest cross-domain accuracy and unknown-detection AUROC, together with the lowest selective risk at nominal 80% coverage. CLIP ViT-B/16 achieved the lowest calibrated ECE. At the same time, the visual summary makes clear that these gains remain incremental rather than decisive. The best frozen-representation accuracy remained below 44%, and even the strongest backbone–score combination left high residual risk at operationally relevant coverage levels. The reliability gap narrows with stronger backbones but does not close.

### 3.5 Ensemble baselines

Two ensemble baselines were constructed to assess whether model averaging can narrow the reliability gap beyond single-model results. The primary baseline is a warm-start end-to-end ResNet50 ensemble (one root model plus four bootstrap-resampled warm-start members), which provides genuine weight-space diversity through independent gradient trajectories. The supplementary baseline is a lightweight DINOv2-S/14 linear-probe ensemble (five linear heads on shared frozen features), which enables uncertainty decomposition but is acknowledged as having limited diversity because all members share the same backbone representation.

**Figure 7:**
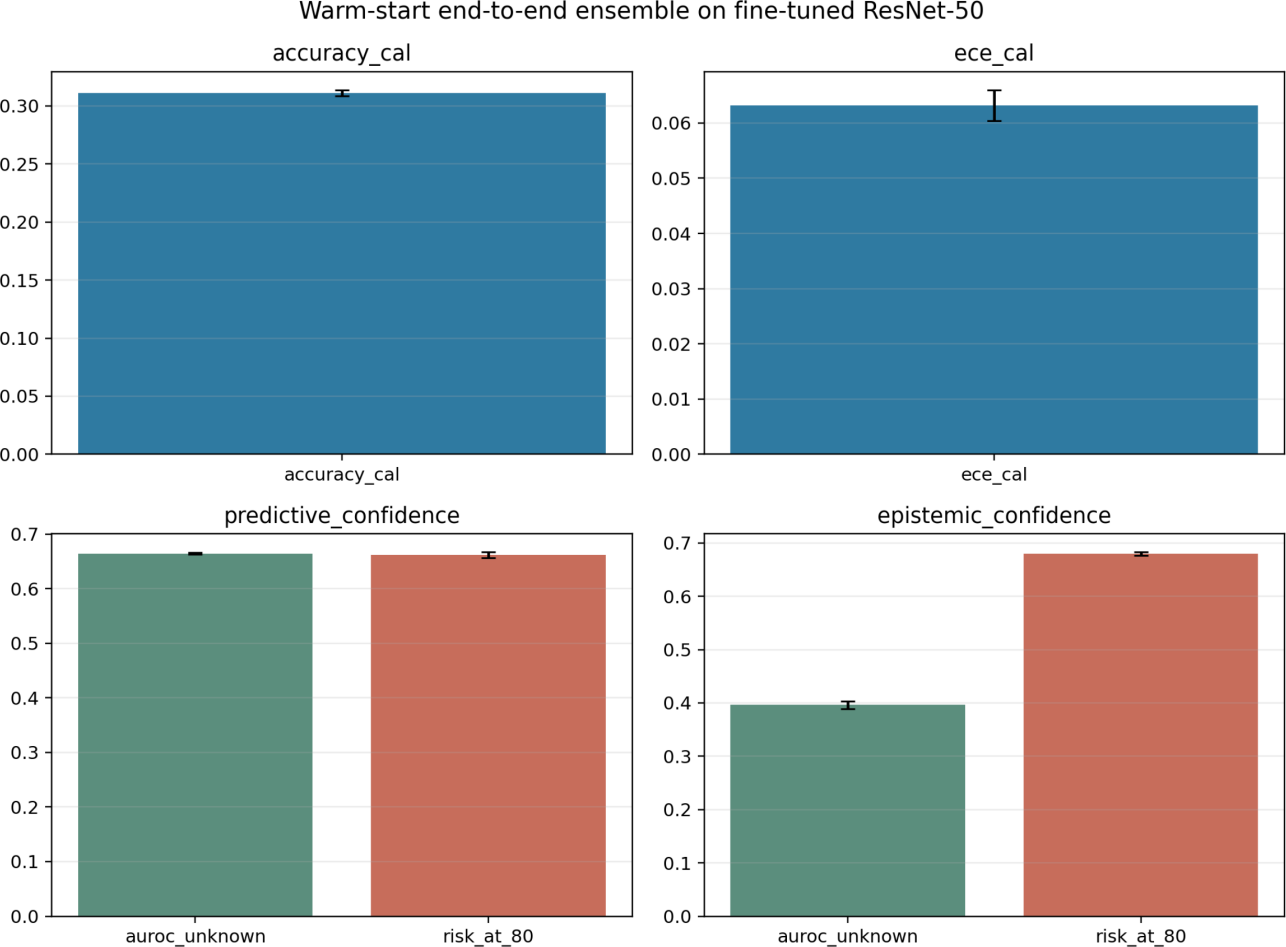
Key deployment metrics for the warm-start end-to-end ResNet-50 ensemble under repeated grouped setting B evaluation. Despite substantial calibration improvement (ECE reduced from 0.476 to 0.063), accuracy and unknown-detection performance remain comparable to the single fine-tuned baseline.

Under repeated grouped Setting B evaluation [mean **T*_*B*_*= 4.10; 95% CI (3.94, 4.26)], the warm-start ensemble achieved a mean accuracy of 31.1% [95% CI (30.9%, 31.3%)] and macro-F1 of 0.279—comparable to the single fine-tuned ResNet-50 baseline (32.1%), as expected given the warm-start initialization from the same root model. The most consequential effect of ensemble averaging was on calibration: raw ECE of 0.476 fell to 0.063 after temperature scaling, an 87% relative reduction, and calibrated Brier score was 0.845. For unknown-class detection, the best performing score was calibrated aleatoric confidence, which achieved a mean AUROC of 0.666 [95% CI (0.665, 0.667)], FPR95 of 0.845, and selective risk at nominal 80% coverage of 0.662 [95% CI (0.657, 0.666)]. **Figure 7** summarizes the key deployment metrics for the warm-start end-to-end ResNet-50 ensemble.

The uncertainty decomposition, however, did not follow the expected epistemic pattern. **Table 9** (upper panel) reports the raw ensemble uncertainty components. The mean epistemic mutual information for unknown-class samples (0.0184) was *lower* than for known-class samples (0.0205)—the opposite of what would be expected if category novelty systematically elevated model disagreement. Predictive entropy and the aleatoric proxy were both higher for unknown samples (0.238 vs. 0.199 and 0.220 vs. 0.179, respectively), indicating that the total uncertainty increase on unknown-class inputs was driven entirely by the aleatoric component rather than by inter-member disagreement. The calibrated epistemic confidence score confirmed this inversion, achieving an AUROC of only 0.397—below chance, indicating that epistemic MI was anti-correlated with unknown status in this ensemble.

**Table 9:** Ensemble uncertainty decomposition for both ensemble baselines: known vs. unknown class samples in repeated grouped setting B.

Ensemble	Uncertainty component	Known	Unknown
Warm-start ResNet-50	Predictive entropy	0.1991	0.2379
	Aleatoric proxy (mean member entropy)	0.1785	0.2196
	Epistemic proxy (mutual information)	0.0205	**0.0184**
DINOv2-S/14 linear-probe	Predictive entropy	0.1468	0.2339
	Aleatoric proxy (mean member entropy)	0.1258	0.1922
	Epistemic proxy (mutual information)	0.0210	**0.0417**

Values are computed from raw (uncalibrated) ensemble outputs. The warm-start ResNet-50 ensemble shows an inverted epistemic pattern (unknown MI *<* known MI), while the DINOv2 linear-probe ensemble shows the expected elevation—but its shared frozen backbone limits the interpretive scope of the decomposition.

Bold values indicate the unknown-class epistemic proxy (mutual information) for each ensemble, highlighted to show the epistemic response to unknown-class samples.

The lightweight DINOv2 ensemble achieved higher accuracy [43.8%; 95% CI (43.6%, 44.0%)] and stronger calibration (ECE 0.053, Brier 0.711) than the warm-start ResNet ensemble, reflecting the superior backbone rather than ensemble diversity. After calibration, the predictive entropy confidence score achieved a mean unknown-detection AUROC of 0.779 [95% CI (0.777, 0.782)] and selective risk at nominal 80% coverage of 0.517—the only configuration in this study to surpass the single-model DINOv2 energy baseline (AUROC 0.764, risk@80 0.520) on both metrics simultaneously. The uncertainty decomposition (**Table 9**, lower panel) showed the expected pattern: epistemic MI was approximately twice as large for unknown-class samples (0.0417) as for known-class samples (0.0210). However, because all five members share the same frozen DINOv2 backbone, this decomposition captures only classification-head-level disagreement, not the full model-level epistemic uncertainty that a diverse end-to-end ensemble would provide.

Both ensembles demonstrate that model averaging dramatically improves *post-hoc* calibration under domain shift: calibrated ECE dropped below 0.07 for both configurations, compared to 0.27–0.37 for the best single-model baselines. This calibration improvement is the most robust ensemble contribution observed in this study. However, the two ensembles diverge on the epistemic question. The warm-start ResNet ensemble—which provides genuinely diverse weight-space trajectories—does not show elevated epistemic MI on unknown samples, while the DINOv2 ensemble—which shares a frozen backbone—does. One interpretation is that the DINOv2 result captures category-level ambiguity in the classification heads rather than deep epistemic uncertainty; another is that the warm-start ensemble’s limited accuracy (31%) produces an uncertainty landscape too noisy for the epistemic component to cleanly separate known from unknown categories. Neither interpretation supports a strong epistemic-based rejection strategy. Operationally, neither ensemble closes the deployment gap: the best ensemble-derived selective risk at nominal 80% coverage (0.517, DINOv2) still exceeds 50%, and the warm-start ensemble’s risk@80 of 0.662 is comparable to the unadapted single-model baseline.

### 3.6 Cross-method synthesis

**Table 10** consolidates deployment-relevant metrics across all methods evaluated in this study. Several patterns emerge. First, accuracy improvements from simple target-aware mitigations (AdaBN, moment matching) are genuine but modest, and DANN-style adversarial adaptation degrades performance. Second, representation quality has a larger effect than any *post-hoc* or lightweight adaptation technique: the DINOv2-S/14 frozen backbone raises accuracy from 0.32 to 0.43 and unknown-detection AUROC from 0.61 to 0.76, gains that exceed anything achieved through calibration, scoring, or simple adaptation on the ResNet-50 baseline. Third, ensemble averaging provides the strongest calibration improvement (ECE *<* 0.07), but this does not translate into commensurate gains in accuracy or selective risk. Fourth, no configuration achieves selective risk below 0.50 at nominal 80% coverage, and all configurations summarized in **Table 10** leave FPR95 above 0.78, confirming that the reliability gap narrows incrementally across the full intervention ladder but does not close under any method tested.

**Table 10:** Cross-method synthesis of deployment-relevant metrics in repeated grouped setting B.

Method	Accuracy	ECE_cal_	Brier_cal_	AUROC_unk_	AUPR_unk_	Risk@80%	Risk@50%
Fine-tuned ResNet-50 (MSP)	0.321	0.365	1.005	0.583	0.247	0.643	0.594
+ entropy scoring	—	—	—	0.610	0.261	0.646	0.593
+ AdaBN	0.343	0.288	0.911	0.611	0.260	0.623	0.553
+ moment matching	0.366	0.310	0.925	0.648	0.289	0.594	0.529
+ DANN	0.252	0.245	0.975	0.577	0.251	0.717	0.645
DINOv2-S/14 + energy	0.432	0.309	0.830	0.764	0.452	0.520	0.474
DINOv2-S/14 ensemble (cal.)	0.438	0.053	0.711	0.779	0.479	0.517	0.467
Warm-start ResNet-50 ensemble (cal.)	0.311	0.063	0.845	0.666	0.287	0.662	0.616

Accuracy, ECE, and Brier score reflect calibrated model outputs; AUROC, AUPR, and selective risk use the best performing scoring function for each method. All values are means over 10 repeated grouped splits. Methods are ordered by intervention complexity.

## 4 Discussion

### 4.1 Severity and sources of the reliability gap

The 67.7-percentage-point accuracy drop observed when moving from PlantVillage to PlantDoc reflects a fundamental mismatch between the visual regularities the model has internalized and the heterogeneous conditions it encounters in the field. Controlled-environment datasets encourage reliance on background texture and illumination cues that correlate spuriously with disease labels but carry no diagnostic meaning, inflating in-domain accuracy while undermining generalization (Torralba and Efros, 2011; Barbedo, 2018). What makes this finding especially consequential is that the accuracy collapse is accompanied by persistent overconfidence: mean predicted confidence on PlantDoc remained at 79.76% despite an accuracy of only 32.05%. This combination of poor accuracy and misplaced certainty is arguably more dangerous than poor accuracy alone, because a farmer receiving such a diagnosis would have no reliable signal that the output should be questioned.

The shift quantification and Grad-CAM analyses reported in Section 3.2 provide concrete evidence for why this gap is so severe. Large-effect-size differences in image-level statistics—saturation (*d* = 3.90), border edge density (*d* = 3.33), and foreground-occupancy proxy (*d* = 2.48)—establish that PlantVillage and PlantDoc occupy substantially different regions of visual feature space. Grad-CAM visualizations on matched source–target pairs reveal the operational consequence: the model’s attention shifts from central lesion regions in controlled images to background-dominated or border-adjacent areas in field images, even when producing high-confidence predictions. These findings transform the reliability gap from an abstract accuracy number into a mechanistically interpretable phenomenon: the model has learned to exploit visual regularities present in controlled environments but absent—or inverted—under field conditions.

### 4.2 How much do standard mitigations help?

The backbone comparison directly addresses whether the reliability gap is an artifact of a single architecture. Under the frozen-representation protocol with five backbones spanning three pretraining paradigms—ImageNet-supervised CNN (ResNet-50), ImageNet-supervised transformer (ViT-S/16), self-supervised transformer (DINOv2-S/14), and vision–language models (CLIP ViTB/16, CLIP ResNet-50)—the qualitative conclusion persists. DINOv2-S/14 raised cross-domain accuracy from 28% to 43% and unknown-detection AUROC from 0.68 to 0.76, improvements that are both statistically significant (paired Wilcoxon *p _<_*0.005) and practically meaningful. CLIP ViT-B/16 achieved competitive accuracy (41.4%) but lower unknown-detection performance (AUROC 0.628), while CLIP ResNet-50 performed comparably to frozen ImageNet ResNet-50, suggesting that vision–language pre-training does not automatically improve cross-domain reliability when paired with a CNN architecture. Yet even the most favorable backbone–score combination left selective risk at nominal 80% coverage above 0.51 and FPR95 above 0.81.

An important distinction must be drawn between statistical significance and practical adequacy. The paired Wilcoxon tests confirm that backbone differences are robust and reproducible—this is valuable evidence that representation quality matters materially. But statistical significance does not imply operational sufficiency. An accuracy improvement from 0.321 to 0.432 is genuine, yet risk@80 remaining above 0.51 means that the best single-model configuration still misclassifies more than half the samples it chooses to answer at operationally relevant coverage levels. These improvements narrow the gap; they do not close it.

Simple target-aware adaptation baselines further refine this picture. Feature moment matching improved ResNet-50 accuracy from 0.321 to 0.366 and reduced risk@80 from 0.643 to 0.594—the strongest simple-adaptation result. AdaBN provided more modest gains (accuracy 0.343, risk@80 0.623), while DANN-style adversarial adaptation degraded performance below the unadapted baseline (accuracy 0.252), suggesting that adversarial feature alignment can disrupt task-relevant discriminative structure under heterogeneous domain shift. The overall pattern confirms that simple target-aware corrections help and should be applied where target-domain data is available, but they do not transform the reliability profile into one suitable for autonomous deployment.

Temperature scaling addresses overconfidence with minimal overhead, and our results reveal an important asymmetry between calibration settings. Under Setting A, where the temperature is tuned on source-domain validation data, the ECE improvement is modest (7.8% relative reduction). Under the grouped Setting B protocol, the improvement is substantially larger (mean calibrated ECE 0.365 across 10 repeats). Yet this stronger calibration did not translate into low selective risk at useful coverage. Small target-domain calibration sets should therefore be viewed as helpful for confidence alignment, not as sufficient remedies for deployment risk.

Selective prediction complements calibration by offering an explicit mechanism for quantifying residual risk. The coverage–risk curves show that the main benefit of abstention in this study is diagnostic rather than operational: it exposes how unstable confidence thresholds become under domain shift and how limited the attainable low-risk operating points remain. The stronger score baseline comparison sharpened this point rather than weakening it: energy and entropy improved ranking quality modestly, but even when extended to the strongest DINOv2 backbone, energy scoring reduced risk@80 only to 0.52—a genuine improvement that still falls well short of the levels required for autonomous operation.

### 4.3 Ensemble calibration and epistemic uncertainty

The two ensemble baselines demonstrate that model averaging provides the strongest calibration improvement observed in this study. Both the warm-start ResNet-50 ensemble (ECE 0.063) and the DINOv2 linear-probe ensemble (ECE 0.053) achieved calibrated ECE values well below any single-model baseline (*>*0.27), indicating that ensemble logit averaging produces a geometry far more amenable to *post-hoc* temperature correction. This calibration advantage is the most robust and practically relevant ensemble contribution.

The epistemic uncertainty decomposition, however, yields a mixed picture that must be reported honestly. The DINOv2 linear-probe ensemble showed the expected pattern—epistemic MI approximately twice as large for unknown-class samples (0.0417) as for known-class samples (0.0210)—consistent with category novelty elevating model-level disagreement. But the warm-start ResNet-50 ensemble, which provides genuinely diverse weight-space trajectories through independent gradient paths from a shared root, showed the *opposite*: unknown MI (0.0184) was lower than known MI (0.0205), and the calibrated epistemic confidence score achieved an AUROC of only 0.397—below chance. Two interpretations merit consideration. First, the DINOv2 result may reflect classification-head-level ambiguity on a shared frozen representation rather than deep epistemic uncertainty; the 2:1 MI ratio, while real, captures a narrower form of disagreement than a full deep ensemble would provide. Second, the warm-start ensemble’s limited accuracy (31%) may produce an overall uncertainty landscape too noisy for the epistemic component to cleanly separate known from unknown categories. Neither interpretation supports a strong epistemic-based rejection strategy, and neither ensemble should be presented as providing definitive evidence about the epistemic structure of the reliability gap.

Operationally, the calibrated DINOv2 ensemble achieved the highest unknown-detection AUROC in this study (0.779) and the lowest selective risk at nominal 80% coverage (0.517), surpassing the single-model DINOv2 energy baseline on both metrics. These gains are genuine but incremental, and risk@80 above 0.50 reinforces that even the strongest configuration falls short of autonomous deployment reliability.

### 4.4 Limitations and future work

Several limitations should be acknowledged. The evaluation is confined to two datasets—PlantVillage and PlantDoc—and 21 shared disease categories within a single source–target scenario. Although representative of a controlled-to-field domain shift, this pair does not capture the full diversity of crops, diseases, and imaging conditions in global agriculture. The target task is leaf-level classification on PlantDoc crop images, not end-to-end diagnosis from raw field photographs; errors from localization, segmentation, or scene-level clutter are not quantified. Extending the evaluation to additional corpora (e.g., PDDR, PlantCLEF) and end-to-end pipelines would strengthen external validity.

At the architectural level, the backbone comparison now spans five architectures across three pre-training paradigms, including two CLIP variants, providing substantially stronger evidence that reliability conclusions are not contingent on a single architecture. However, the comparison employed a frozen-representation protocol with linear probes rather than full end-to-end fine-tuning. While this isolates representation quality from optimization confounds, it does not characterize the reliability properties of fully fine-tuned transformers or foundation models, which may exhibit qualitatively different calibration behavior. The comparison also does not cover compound-scaled CNN families such as EfficientNet (Tan and Le, 2019). The warm-start ResNet-50 ensemble uses end-to-end fine-tuning but starts from a shared root checkpoint, limiting weight-space diversity relative to independently trained cold-start members; the DINOv2 ensemble shares a frozen backbone entirely. More expressive ensemble or Bayesian methods (Gal and Ghahramani, 2016; Lakshminarayanan et al., 2017; Sensoy et al., 2018) may capture uncertainty structure that these baselines cannot.

At the methodological level, the adaptation baselines (AdaBN, moment matching, DANN) test only lightweight domain-alignment techniques applied to the ResNet-50 case study. More sophisticated adaptation methods—including deeper feature alignment, optimal transport, or multisource adaptation—and their interaction with stronger backbones remain unexplored. The results should therefore be interpreted as a strengthened baseline study establishing the severity and persistence of the gap, not as an exhaustive search over possible mitigations.

Regarding the robustness analysis, we repeated the grouped 10% calibration split 10 times and reported 95% confidence intervals for split-sensitive metrics. The parent-image-aware grouping constraint was retained as a methodological safeguard against data leakage, even though it did not change the actual data allocation in the current PlantDoc version. Richer acquisition-event metadata and broader multi-site target datasets would support a more comprehensive variance analysis.

Two broader considerations merit attention. First, real-world deployment unfolds over time; seasonal variation, emerging disease strains, and hardware changes may cause additional drift, requiring continuous calibration monitoring. Second, the evaluation framework focuses exclusively on technical metrics and does not assess how end users—farmers, agronomists, extension workers— respond to calibrated confidence scores and abstention signals. Field trials measuring user trust and decision-making behavior would provide the critical human-centered evidence needed to translate technical improvements into real-world impact.

### 4.5 Broader context and recommendations

The challenges documented here are symptomatic of a broader tension in applied machine learning between benchmark performance and deployment reliability—extensively discussed in medical imaging and autonomous driving, but comparatively under-examined in agricultural AI. Several recommendations follow.

At the level of experimental reporting, publications evaluating plant disease classifiers should routinely present calibration error, Brier scores, and selective risk alongside accuracy and F1, particularly under domain shift. Deployment claims should be substantiated with evaluation on field-photograph corpora rather than relying exclusively on controlled-environment benchmarks.

At the level of system design, abstention should not be assumed to confer safe automation merely because it improves selected metrics. Where selective prediction is considered, its achieved coverage and residual risk should be reported jointly and interpreted conservatively. The finding that these limitations persist across five backbone architectures, three adaptation baselines, and two ensemble configurations reinforces the generality of this caution.

At the level of deployment practice, *post-hoc* calibration and simple target-aware adaptation are worth applying because they are inexpensive and improve confidence alignment, but the present results show that such gains alone do not establish operational readiness. Representation quality materially affects all deployment-relevant dimensions, and this should inform architecture selection in practice. Any credible deployment assessment must evaluate selective risk, threshold-transfer stability, and behavior on categories absent from training before autonomous operation is considered.

## 5 Conclusion

This study has characterized the reliability gap in cross-domain plant disease classification through systematic benchmarking of standard mitigation techniques under a controlled-to-field shift. Evaluating models trained on PlantVillage against parent-image-audited PlantDoc leaf crops, we documented a triad of deployment risks: severe accuracy collapse (67.7-percentage-point drop), persistent overconfidence, and limited ability to reject unknown disease categories. Quantitative shift analysis and Grad-CAM visualizations established that this gap is driven by identifiable visual distribution differences that cause models to attend to spurious rather than diagnostically relevant features.

A comprehensive mitigation ladder was evaluated. Simple target-aware adaptations (AdaBN, moment matching) provided genuine but limited improvements, while DANN-style adversarial adaptation degraded performance. Extending the analysis across five backbone architectures— including two CLIP variants—under a standardized frozen-representation protocol confirmed that stronger representations, particularly self-supervised DINOv2-S/14, narrow all dimensions of the gap, with all accuracy and macro-F1 pairwise differences confirmed as statistically significant. Two ensemble baselines demonstrated that model averaging dramatically improves calibration (ECE reduced to <0.07), but the uncertainty decomposition yielded mixed results: the DINOv2 linear-probe ensemble showed elevated epistemic MI on unknown samples, while the warm-start ResNet-50 ensemble—which provides greater weight-space diversity—did not, precluding a strong epistemic-based rejection claim. Even the strongest overall configuration (calibrated DINOv2 ensemble) left selective risk at nominal 80% coverage above 0.50.

The central lesson is that benchmark accuracy alone is an inadequate target for evaluating agricultural diagnostic models. The present work contributes a reproducible evaluation protocol together with multi-backbone, multi-adaptation, and ensemble-level evidence showing that the reliability gap under controlled-to-field shift is severe, mechanistically interpretable, and only partially reducible by standard techniques. Future work should investigate richer domain adaptation strategies, full deep ensembles with independently trained members, additional source–target scenarios, and field trials with end users before stronger deployment claims are warranted.

## Data Availability

The datasets analyzed in this study are publicly available from their original sources: PlantVillage (https://github.com/spMohanty/PlantVillage-Dataset) and PlantDoc (https://github.com/pratikkayal/PlantDoc-Dataset). The processed files supporting the findings of this study, including the category alignment table and data split manifests used for the analyzed snapshot, are available from the corresponding author upon reasonable request. The code used for data processing, model evaluation, calibration, and abstention analysis is available from the corresponding author upon reasonable request.

## Supplementary material

Supplementary Table 1 Category alignment between PlantVillage and PlantDoc labels, indicating the shared known classes retained for cross-domain evaluation and the PlantDoc categories treated as unknown classes.
